# Comparison Between Intraperitoneal and Intravenous Lidocaine for Postoperative Analgesia After Elective Abdominal Hysterectomy, a Double-Blind Placebo Controlled Study

**Published:** 2015-11

**Authors:** Saghar Samimi, Arman Taheri, Fatemeh Davari Tanha

**Affiliations:** 1 Department of Anesthesiology, Women’s Hospital, Tehran University of Medical Sciences, Tehran, Iran; 2 Department of Anesthesiology, Amir Aelam Hospital, Tehran University of Medical Sciences, Tehran, Iran; 3 Department of Obstetrics and Gynecology, Women’s Hospital, Tehran University of Medical Sciences, Tehran, Iran

**Keywords:** Intravenous, lidocaine, Intraperitoneal, Abdominal Hysterectomy, Pain, Post Operative, lidocaine Adverse Effects

## Abstract

**Objective:** To compare the efficacy of intravenous and intraperitoneal injection of lidocaine and normal saline in relieving postoperative pain after elective abdominal hysterectomy.

**Materials and methods:** For this double-blind randomized controlled study 109 patients undergoing elective abdominal hysterectomy were randomly allocated to three groups :1) IV group (intravenous injection group) received intravenous lidocaine %2 bolus 1.5mg/kg 30 min before incision and then a continuous lidocaine infusion of 2mg/kg and before the wound closure an intraperitoneal injection of N/S , 2) IP group (intraperitoneal group) received intravenous N/S and intraperitoneal lidocaine 3mg/kg , 3) P group (placebo, N/S) received both intravenous and intraperitoneal N/S. The pain scores (VAS) at rest, total morphine consumption , the time to first need for rescue analgesic ,incidence of lidocaine related adverse effects and nausea and vomiting were recorded at 0,2,4,8,12 and 24 hrs postoperatively.

**Results:** The VAS scores were significantly lower in IP and IV groups compared with placebo (p = 0.001). Total consumption of morphine (p = 0.001) and time to firs request of recue analgesic (p = 0.001) were lower too in IP and IV groups.Incidence of vomiting was comparable between groups (p < 0.05) but nausea was higher in control group (p > 0.05).There were not notable lidocaine-related adverse effects. IP and IV groups were not statistically different for all investigated variables.

**Conclusion:** This study showed lidocaine administration both intravenously and intraperitoneally are effective in reducing the postoperative pain and also have opioid sparing effect and can be safely used in elective abdominal hysterectomy without any major adverse effects.

## Introduction

Today various therapeutic protocols are available for management of pain but preventing and relieving the postoperative pain remains an important challenge (1). It is thought that the postoperative pain is inadequately treated in approximately one half of all surgical procedures (2).

Clinical trials suggest a high quality postoperative pain management improves recovery and also reduces the risk of postoperative acute adverse effects (i.e., pulmonary dysfunction), and chronic adverse effect (i.e., delayed recovery and hospital discharge and chronic pain) after various procedures including hysterectomy (3, 4). The abdominal hysterectomy is one of the most common gynecological operations in women, which is associated with severe postoperative pain. Although opioids are usually the gold standard analgesic for major abdominal surgery, but they have many unwanted side effects too, including nausea and vomiting, gastrointestinal symptoms and respiratory depression (5, 6). Therefore a multimodal approach, using local anesthetics may help to improving the quality of analgesia, recovery and reduce opioid dose requirement and side effects.

Lidocaine is an amid local anesthetic that has been used from 1960s, in clinical practices for a wide range of clinical situations (i.e., antiarrhythmia, regional analgesia, treatment of neuropathic pain and central pain and postoperative analgesia (7). It is a Na⁺⁺ channel blocker that can block peripheral hyperexcitability because the mechanism of this phenomenon is partly due to an accumulation of Na channels on the site of damage. On the other hand it reduces the development of postoperation central hyperalgesia by blocking Na⁺⁺ channels on nerve endings of mechanoreceptors in the spinal cord and dorsal root ganglion and suppresses neuronal excitability (8). Lidocaine also reduces visceral pain by inhibition of spinal visceromotor neurons. Local anesthetics have anti-inflammatory effects thus prevent tissue damage in postoperative period (9, 10). Some researchers suggest intravenous lidocaine results in prompting the return of bowel function, facilitating rehabilitation and decreases postoperative hospital stay (11, 12). The mechanisms of effect of intraperitoneal local anesthetics is probably blocking of peritoneal free nerve endings and/or systemic absorption of drug (13).

## Materials and methods

For this double-blinded randomized, controlled study, after approving by ethics and clinical studies committee of Tehran University of Medical Sciences (ethics code project: 26072), 130 patients aged 35-65 undergoing elective abdominal hysterectomy were screened and 117 of them were scheduled. All of the patients were ASA class 1, 2 (American society of anesthesiologists).

Inclusion criteria were patients 35-65 yrs undergoing elective abdominal hysterectomy and exclusion criteria included: operation duration more than 3 hrs, diagnosis of cancer, need for additional surgery, substance abuse, chronic pain syndromes, allergy to study medications, severe psychologic, hepatic, renal and cardiac diseases and any incision other than pfansteil.

Patients were randomly allocated to 1 of 3 treatment groups using computer randomization number generation: 1) group IV (intravenous injection group) n = 40, 2) group IP (intraperitoneal injection group) n = 40, and 3) group P (placebo or N/S group) n = 41.The primary outcome was pain intensity at rest during first 24 hours postoperative period. The secondary outcomes were total opioid consumption, the time to first request or need for supplementary opioid.

On the day before the surgery during the preanesthesia consultation, the visual analogue scale (VAS) was explained to the patients (0 as no pain and 10 as worst imaginable pain), by an anesthesiologist and written informed consent was obtained.

The study medications were prepared by an anesthesiologist who did not otherwise participated in the study based on patient′s weight and then were enveloped and sealed and patient′s code was recorded on it. The envelopes were opened in operation room 1 hour before starting of induction of anesthesia by an anesthesiologist who was blinded to patient′s study group and type of solution. All of the nurses and patients were blinded to the type of solution and to the patient′s study group allocation. On the day of surgery, after arrival on operation room an intravenous access was established and patients connected to ECG, pulse oximeter and noninvasive blood pressure monitoring. Then they received 2 mg midazolam (Aburaihan Co. Iran Batch No. 2007) intravenously. All patients received standard general anesthesia with propofol (%1 Fresenius Kabi Austria Batch No. 16HF0219) 2 mg/kg, fentanyl (Feniject Aburaihan Co. Iran Batch No. 3002) 2 µg /kg and atracurium (Aburaihan Co. Iran Bach No.0083) 0.5 mg/kg and following the intratracheal intubation they were mechanically ventilated with 50:50 O₂ and N₂O.

For maintenance of anesthesia they received isoflurane (Primal critical care, Inc. USA) 0.8-1.2 MAC, intermittent doses of atracurium and additional fentanyl 1µg/kg if needed to maintain hemodynamic stability during the surgery. At the end of surgery we use from neostigmine (Caspian Tamin Pharmaceutical Co. Iran) 0.05 mg/kg and atropine (Caspian Tamin Pharmaceutical Co. Iran) 0.02 mg/kg for antagonizing neuromuscular block. 

Patients in group IV(intravenous injection group) received lidocaine %2(Aburaihan Co. Iran Batch No.3012) , (1.5 mg/kg bolus injection) 30 minutes before incision and then a continuous lidocaine infusion until 1 hour after the end of surgery and before closure of wound 50 cc N/S intraperitoneally. Patients in group IP (intraperitoneal administration group), received a bolus injection of N/S with equal volume with bolus dose of lidocaine in IV group and then a continuous infusion of N/S and at the end of surgery before the closure of wound they received lidocaine %2, (3 mg/kg) that was diluted with N/S to reach 50 cc volume. Group P(N/S injection group) received a bolus dose of N/S with equal volume with bolus dose of lidocaine in IV group patients and a continuous infusion of N/S until 1 hour postoperatively .At the end of surgery and before wound closure 50 cc N/S was administered into intraperitoneal cavity.

Aftertransferring to the postoperative anesthesia care unit (PACU), all the patients received diclofenac suppository 100 mg.

Patients were assessed in PACU and subsequently in surgical ward for pain intensity using a 10 cm VAS (0 = no pain, 10 = the most imaginable pain), need to rescue analgesic and occurrence of nausea and vomiting at 0, 2, 4, 8, 12, 24hours postoperatively and also each time that the patient complained of pain over first 24 hours after surgery by an assistant who was blinded to the detail of study. All of the data were recorded on a sheet.

Postoperative pain was treated with morphine 2 mg intravenously when the patients asked for an analgesic or her VAS was ≥ 4.

Statistical analysis was performed with SPSS for windows version 18 (SPSS, Chicago, IL).One way analysis of variance (ANOVA) was used for comparison among three groups, also LSD post hoc test was used .Data were expressed as mean (± SD), number, (%), or median. A p value less than 0.05 were considered significant.

## Results

A total of 117 patients who were scheduled for elective abdominal hysterectomy over a period of 24 months were included. During the study, 8 of them excluded from postoperative data analysis : 5 because of the operation was lasted more than 3 hours and 3 because of need to additional procedures, and we completed the study with 109 patients(IV group = 36, IP group = 35 and P group = 38).

There was no significant difference between three groups with regard to age, height, weight, ASA class, also duration of anesthesia and surgery and opioid consumption during the operation (Table 1).

The pain intensity was significantly reduced in both IV and IP groupscompared with control group until 12 hours postoperatively (p = 0.001) and there was no significant difference between IV and IP group in this regard (p > 0.05) (Table 2).The first time of analgesia request in patients receiving N/S was significantly shorter than IV and IP groups (p > 0.05) (Table 3).

**Table 1:** Data of patients and operations

	IV group (n =39)	IP group (n =39)	p group (n =38)	p value
**Age (years)**	**46.2 ± 12.9**	**49.3 ± 10.6**	**48.2 ±11.2**	**0.492**
**Height (cm)**	**157 ± 8.33**	**160 ±7.42**	**158 ± 8.20**	**0.245**
**Weight (Kg)**	**63.3 ± 7.10**	**64.1 ± 4.90**	**62.30 ± 7.18**	**0.476**
**Operation duration (min)**	**95 ± 20.70**	**89 ±25.17**	**92 ± 21.20**	**0.501**
**Anesthesia duration (min)**	**105 ± 23.28**	**102 ± 24.67**	**105 ± 11.04**	**0.762**
**Fentanyl use (µg)**	**239 ± 10.8**	**236 ± 15.3**	**242 ± 11.5**	**0.231**

Values are means ± SD. Value of p less than 0.05 is significant

**Table 2:** Pain scores (VAS) at rest

	IV group (n = 39)	IP group (n = 39)	N/S group (n = 38)	p value IV/p	p value IP/p	p value IV/IP
**VAS (0hr)**	**1.54 ± 0.58**	**1.97 ± 0.52**	**3.41 ± 0.59**	**0.0001**	**0.001**	**0.238**
**VAS (2hrs)**	**3.21 ± 0.86**	**3.45 ± 0.46**	**4.51 ± 0.55**	**0.0001**	**0.001**	**0.130**
**VAS (4hrs)**	**4.13 ± 0.83**	**4.05 ± 0.55**	**5.19 ± 0.66**	**0.0001**	**0.001**	**0.618**
**VAS (8hrs)**	**3.72 ± 0.56**	**4.15 ± 0.76**	**5.50 ± 0.53**	**0.0001**	**0.001**	**0.514**
**VAS (12hrs)**	**4.50 ± 0.51**	**4.50 ± 0.74**	**5.20 ± 0.74**	**0.0001**	**0.001**	**1**
**VAS (24hrs)**	**3.61 ± 0.55**	**3.50 ± 0.70**	**4.01 ± 0.51**	**0.64**	**0.612**	**0.842**

1VS: Visual analogue Scale;2 Value of p less than 0.05 is significant

**Table 3:** The time to first analgesic requirement

	IV group	IP group	N/S group	p value IV/p, IP/P	p value IV/IP
**The time to first analgesic requirement (min)**	**75 ± 12.5**	**62 ± 11.9**	**23 ± 10.4**	**0.0001**	**0.840**

Value of p less than 0.05 is significant

**Table 4:** Morphine consumption in first day after surgery

	IV group	IP group	N/S group	p value IV/p, IP/p	p value IV/IP
**Morphine consumption (mg)**	**17 ± 1.5**	**18 ± 1.2**	**25 ± 2.7**	**0.0001**	**0.761**

Value of p less than 0.05 is significant

**Table 5:** The incidence of nausea and vomiting in first day after surgery

	**IV group** **n = (39)**	**IP group** **n = (39)**	**N/S group** **n = (39)**	**p value**
Nausea [n (%)]	11 (%28)	13 (%33)	21 (%55)	0.036
Vomiting [n (%)]	4 (%10)	4 (%10)	10 (%26)	0.081

Value of p less than 0.05 is significant

During the 24 hours postoperatively morphine consumption was significantly lower in IV and IP groupscompared with control group (P < 0.05) (Table 4). The incidence of vomiting was comparable between three groups but the incidence of nausea was higher in control group compared with two other groups (Table 5).

No significant difference were founded between IP and IV groups in VAS scores, time to first request for rescue analgesic, total consumption of morphine and incidence of nausea and vomiting , also none of the patients experienced lidocaine-related adverse effects.

## Discussion

We have shown that pain following abdominal hysterectomy was better relieved by intraperitoneal and intravenous lidocaine compared with N/S until 12 hours after the end of surgery and also need for postoperative morphine during first day after surgery was reduced (14-16). Several clinical studies have investigated the effect of lidocaine on preventing postoperative pain and there are contradictory results about the efficacy of intravenous and intraperitoneal local anesthetics in treatment of postoperative pain. Some of the researchers suggest these methods are valid and have beneficialeffects (17-19) and some don′t believe in them (20-23). Few studies are performed for local anesthetics other than bupivacaine and most of the studies are in laparoscopic procedures (12, 14, 16, 18, 20-23). It seems the differences between results of studies probably are due to differences in pain evaluation method, type of procedures and the dose and timing of drug administration. The timing of drug administration is very important in drug efficacy and most of investigators suggested that local anesthetics must be administered before the activation of pain pathway by nociceptive stimulation.In our study we administered lidocaine1.5 mg/kg bolus intravenously 30 minutes before incision and a continuous infusion of lidocaine 2 mg/kg/hr until 1 hour after the end of surgery.Kaba et aladministered small dose of lidocaine until 24 hours after surgery, their results were like our data (24). Others, started lidocaine before the surgery following a continuous infusion until 1 hour postoperatively with different doses (25-27). Koppert and colleagues (25) reported that intravenous lidocaine prevents postoperative pain and reduces opioid consumption after major abdominal surgerybut unlike with us, they observed lower postoperative pain intensity in lidocaine group during movement no at rest (24). BK Baral and his colleagues (26) administered lidocaine with a bolus dose like us but the infusion rate was 1.5mg/kg and their results were similar to our study except for, we used morphine as rescue analgesic, therefore unlike them we had no problem with relieving postoperative pain in patients who needed to additional analgesic.Groudine and his coworkers (27) also concluded that in lidocaine group, pain intensity and opioid consumption were significantly lower than control group but unlike us, in their research the time to first rescue analgesic was not different between groups.Jun HeumYon and colleagues administered lidocaine bolus 1.5 mg/kg and then a continuous infusion of lidocaine 2mg/kg/hr until the end of surgery (28). In their study, opioid consumption was lower in lidocaine group for 12 hr that is similar to our result but pain intensity was significantly lower in lidocaine group for 24 hr but in our research pain was significantly lower for 12 hours in lidocaine group compared with control.

Anil Gupta and colleague (29) in 2004 explained that intraperitoneal levobupivacaine was effective in reducing postoperative pain after abdominal hysterectomy only 2 hours after surgery. Waleed El Sherbiny (30) in 2009 used intraperitoneal lidocaine with a different dose compared with us and resulted that it was more effective than normal saline for relieving postoperative pain in patients undergoing gynecological laparoscopy until 4 hours postoperatively. In our study we used 2mg/kg lidocaine which had received to 50 cc volume with N/S and it caused analgesia for 12 hours postoperatively.

Conclusion: We have demonstrated, using of lidocaine both intravenously and intraperitoneally are effective and safe for relieving pain after elective abdominal hysterectomy.These methods reduce opioid consumption in first day after surgery too.

The limitation of our study was that we studied only the women undergoing elective abdominal hysterectomy and evaluated pain at rest thus our results may not attributable to: pain intensity at movement, men, emergency surgeries and other types of procedures, therefore more studies are needed.
